# Supplementary material for: Lung injury-induced activated endothelial cell states persist in aging-associated progressive fibrosis
Source: Nat Commun. 2024 Jun 27;15:5449. doi: 10.1038/s41467-024-49545-x (PMC11211333; doi:10.1038/s41467-024-49545-x)
Supplement: Supplementary file 1 — Supplementary Information [file 41467_2024_49545_MOESM1_ESM.pdf]

Lung injury-induced endothelial cell states persist in aging-associated progressive fibrosis.

Ahmed A. Raslan[1,2,3], Tho X. Pham[1,2], Jisu Lee[1], Konstantinos Kontodimas[2,4], Andrew Tilston-Lunel[2,4], Jillian Schmottlach[1], Jeongmin Hong[1,2], Taha Dinc[1], Andreea M. Bujor[1], Nunzia Caporarello[5], Aude Thiriot[6], Ulrich H. von Andrian[6,7], Steven K. Huang[8], Roberto F. Nicosia[9], Maria Trojanowska[1,2], Xaralabos Varelas[2,4], Giovanni Ligresti[1,2].

Affiliations:

[1]Department of Medicine, Boston University Chobanian and Avedisian School of Medicine, Boston University, Boston, MA, USA
[2]Pulmonary Center, Boston University Chobanian and Avedisian School of Medicine, Boston, MA, USA
[3]Department of Zoology, Faculty of Science, Assiut University, Assiut, Egypt
[4]Department of Biochemistry and Cell Biology, Boston University Chobanian and Avedisian School of Medicine, Boston, MA, USA
[5]Department of Medicine, Loyola University Chicago, Chicago, IL, USA
[6]Department of Immunology, Harvard Medical School, Boston, MA, USA
[7]The Ragon Institute of MGH, MIT and Harvard, Cambridge, MA, USA
[8]Department of Internal Medicine, University of Michigan Medical School, Ann Arbor, MI, USA
[9]Department of Laboratory Medicine and Pathology, University of Washington, Seattle, WA, USA

Corresponding author:

Giovanni Ligresti, PhD. Department of Medicine, Division of Rheumatology, Arthritis and Autoimmune Diseases Research Center, Boston University Chobanian & Avedisian School of Medicine, Boston, MA, 02118, USA. Phone: 617.358.6786; Email: ligresti@bu.edu.

Xaralabos Varelas, PhD. Department of Biochemistry and Cell Biology, Boston University Chobanian & Avedisian School of Medicine, Boston, MA 02118, USA. Phone: 617.358.4575; Email: xvarelas@bu.edu

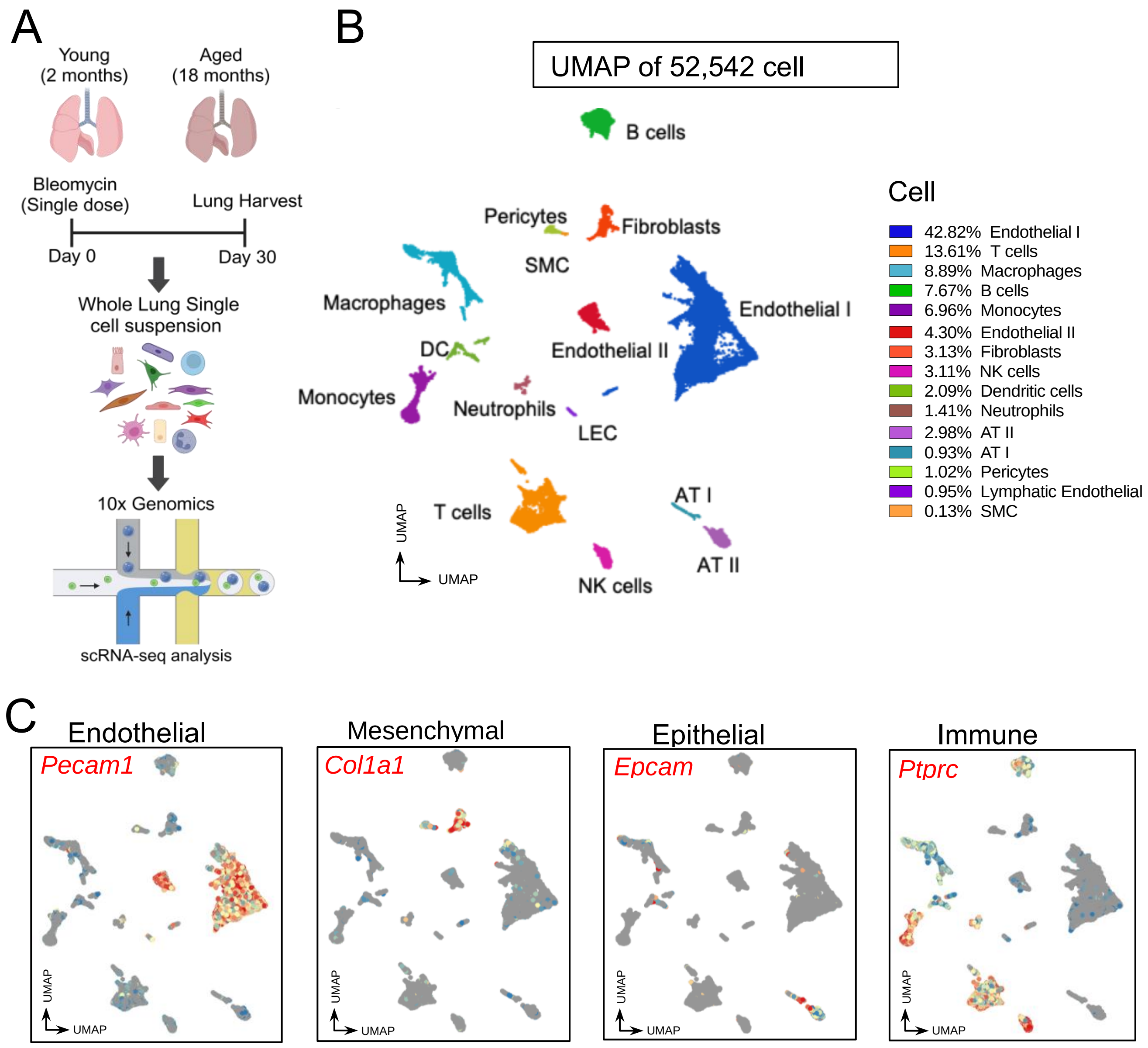

Supplementary Figure 1. scRNA-seq analysis of young and aged mouse lungs following bleomycin challenge. (A) Young sham (*n* = 1, 11767 cells), aged sham (*n* = 2, 16507 cells), bleomycin-treated young (*n* = 1, 5893 cells), and bleomycin-treated aged (*n* = 2, 18375) lungs were harvested at day 30 post bleomycin delivery and prepared for scRNA-Seq analysis. This schematic was created with BioRender.com. **(B)** UMAP embedded visualization of the cells from

all lung samples shows different cell populations and their composition. **(C)** UMAP plots of single cells showing gene expression for indicated marker genes.

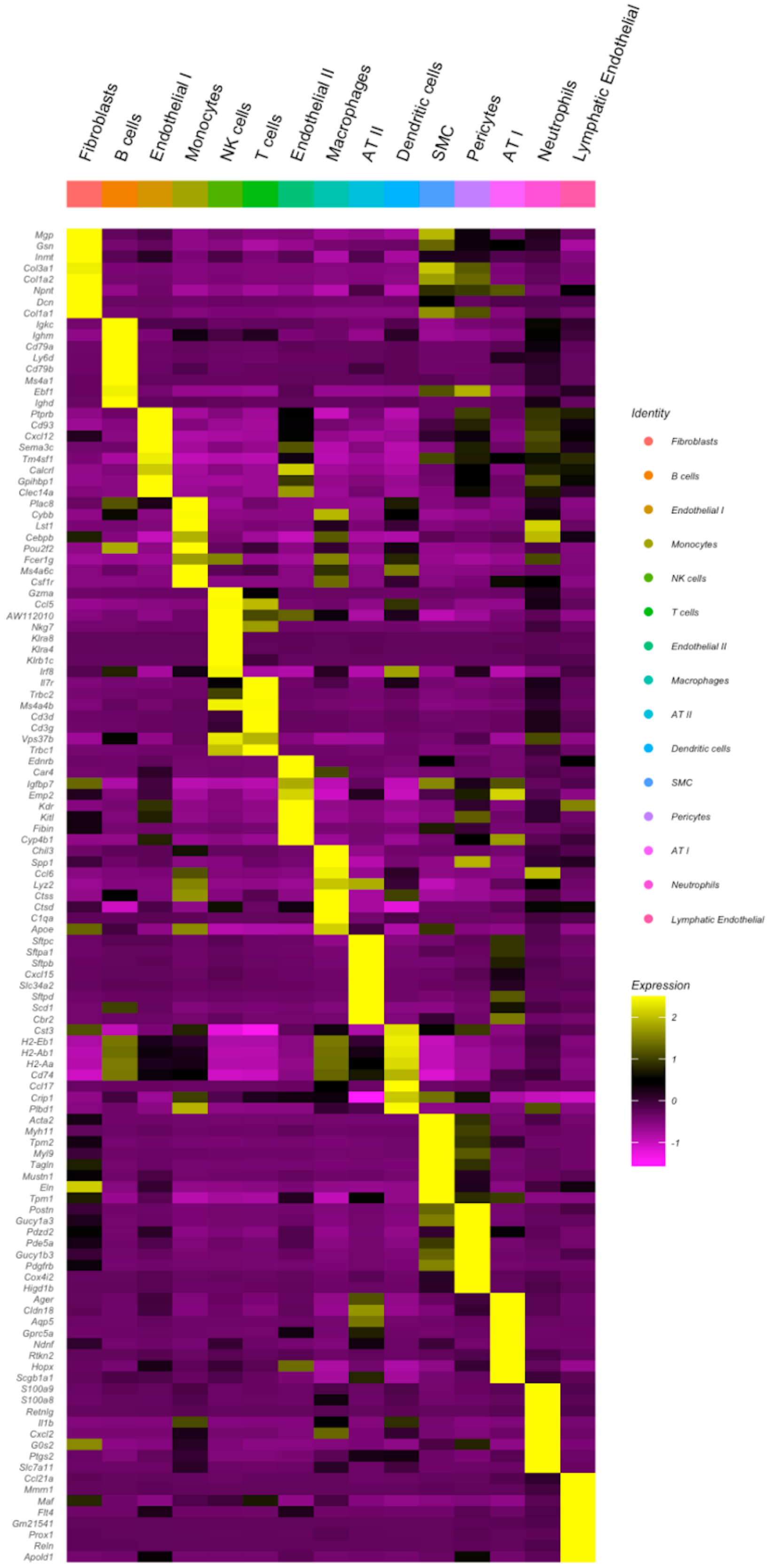
Fibroblasts
B cells
Endothelial I
Monocytes
NK cells
T cells
Endothelial II
Macrophages
AT II
Dendritic cells
SMC
Pericytes
AT I
Neutrophils
Lymphatic Endothelial
Mgp
Gsn
Inmt
Col3a1
Col1a2
Npnt
Dcn
Col1a1
Igkc
Ighm
Cd79a
Ly6d
Cd79b
Ms4a1
Ebf1
Ighd
Ptprb
Cd93
Cxcl12
Sema3c
Tm4sf1
Calcrl
Gpihbp1
Clec14a
Plac8
Cybb
Lst1
Cebpb
Pou2f2
Fcer1g
Ms4a6c
Csf1r
Gzma
Ccl5
AW112010
Nkg7
Klra8
Klra4
Klrb1c
Irf8
Il7r
Trbc2
Ms4a4b
Cd3d
Cd3g
Vps37b
Trbc1
Ednrb
Car4
Igfbp7
Emp2
Kdr
Kitl
Fibin
Cyp4b1
Chil3
Spp1
Ccl6
Lyz2
Ctss
Ctsd
C1qa
Apoe
Sftpc
Sftpa1
Sftpb
Cxcl15
Slc34a2
Sftpd
Scd1
Cbr2
Cst3
H2-Eb1
H2-Ab1
H2-Aa
Cd74
Ccl17
Crip1
Plbd1
Acta2
Myh11
Tpm2
Myl9
Tagln
Mustn1
Eln
Tpm1
Postn
Gucy1a3
Pdzd2
Pde5a
Gucy1b3
Pdgfrb
Cox4i2
Higd1b
Ager
Cldn18
Aqp5
Gprc5a
Ndnf
Rtkn2
Hopx
Scgb1a1
S100a9
S100a8
Retnlg
Il1b
Cxcl2
G0s2
Ptgs2
Slc7a11
Ccl21a
Mmrn1
Maf
Flt4
Gm21541
Prox1
Reln
Apold1
Identity
Fibroblasts
B cells
Endothelial I
Monocytes
NK cells
T cells
Endothelial II
Macrophages
AT II
Dendritic cells
SMC
Pericytes
AT I
Neutrophils
Lymphatic Endothelial
Expression
2
1
0
-1

Supplementary Figure 2. Heat map of marker genes for all identified cell types. Each cell type is defined by the top eight most expressed genes. Each column represents the average expression value for each lung sample, hierarchically grouped by cell type.

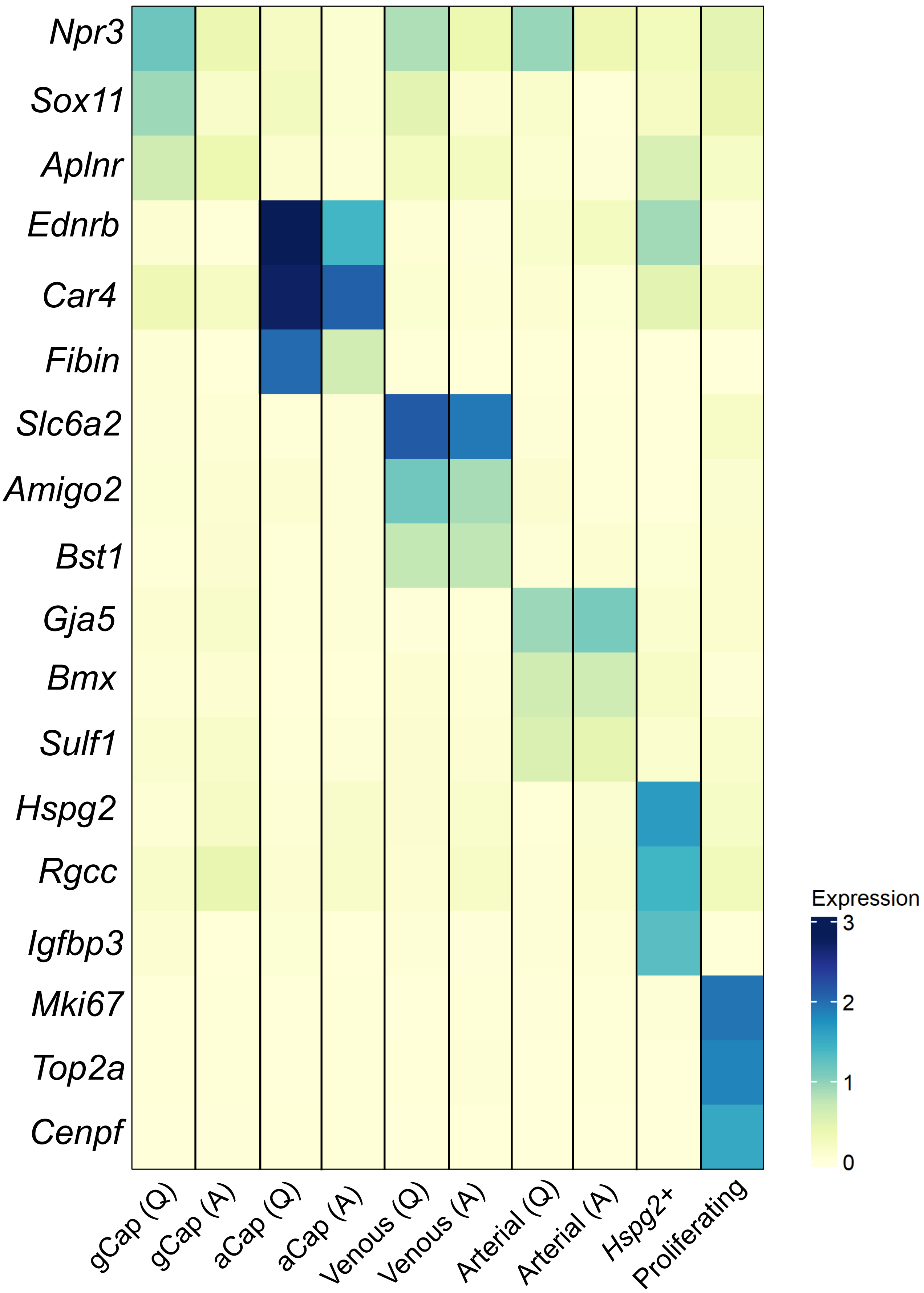

Npr3
Sox11
Aplnr
Ednrb
Car4
Fibin
Slc6a2
Amigo2
Bst1
Gja5
Bmx
Sulf1
Hspg2
Rgcc
Igfbp3
Mki67
Top2a
Cenpf
gCap (Q)
gCap (A)
aCap (Q)
aCap (A)
Venous (Q)
Venous (A)
Arterial (Q)
Arterial (A)
Hspg2+
Proliferating
Expression
3
2
1
0

Supplementary Figure 3. Heat map of marker genes for all endothelial cell types. Each EC lineage is defined by the top three most expressed genes. Each column represents the average expression value for each lung sample, hierarchically grouped by EC type. Quiescent (Q), activated (A).

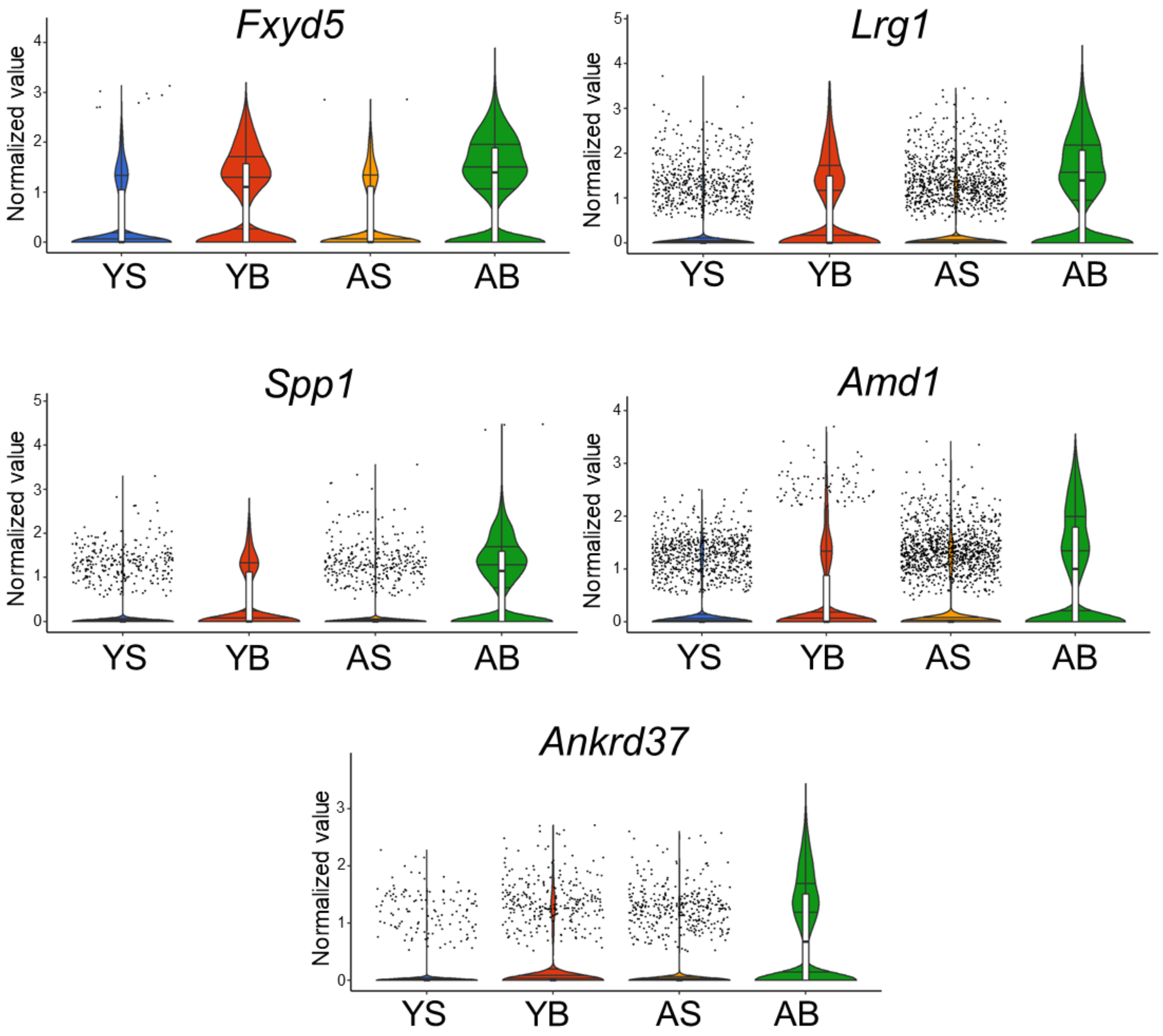

Supplementary Figure 4. Activated EC from aged mouse lung express high level of activation marker genes. Violin plots of the expression of activation marker genes in EC from Young and Aged mouse. Young Sham (YS, *n* = 5717 cells); Young Bleo (YB, *n* = 2211 cells); Aged Sham (AS, *n* = 6821 cells); Aged Bleo (AB, *n* = 9462 cells). Each box plot displays the median value as the center line, the upper and lower box boundaries at the first and third quartiles (25th and 75th percentiles), and the whiskers depict the minimum and maximum values.

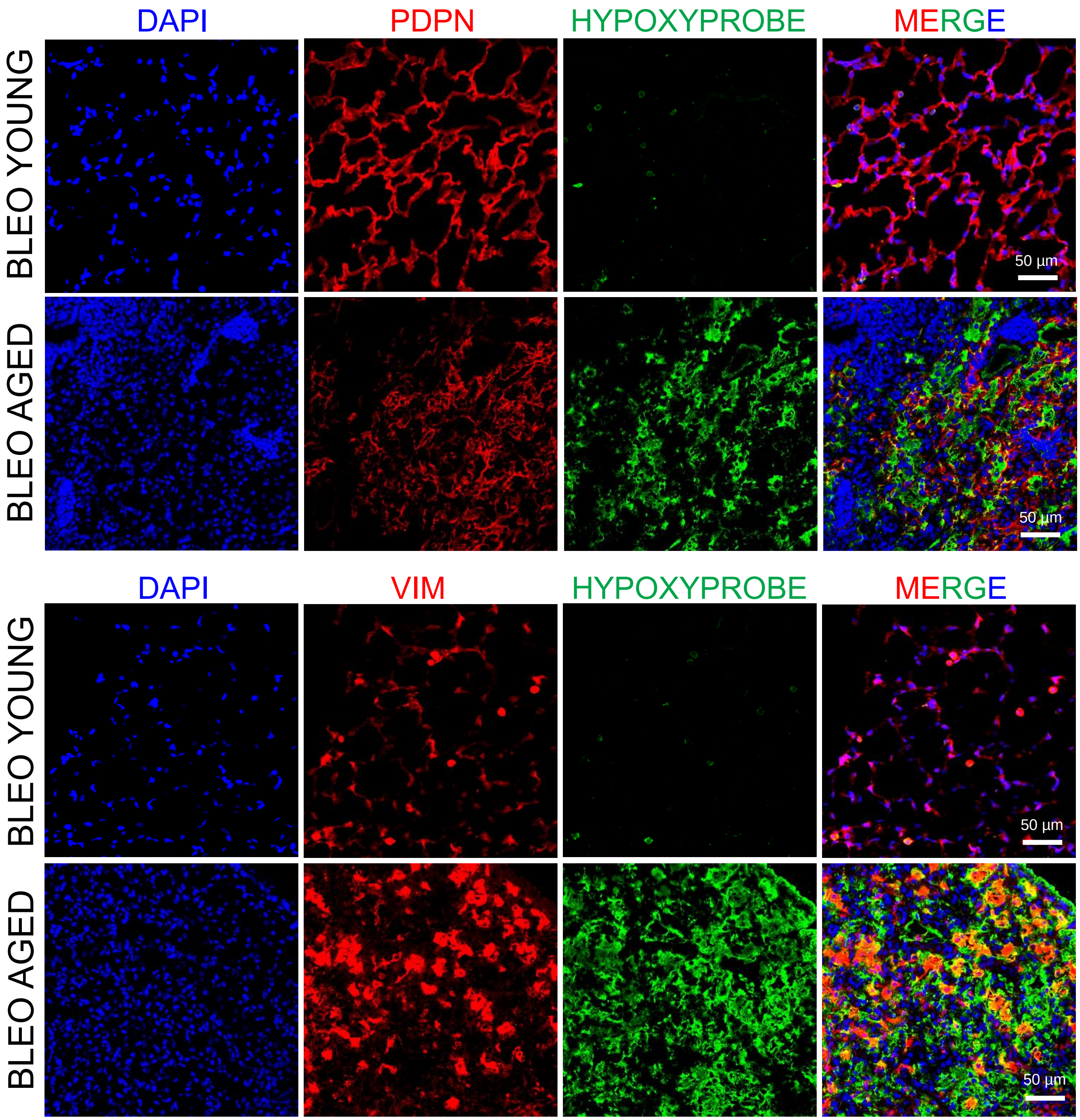

Supplementary Figure 5. Hypoxia in fibrotic aged lungs is detected in cells of epithelial and mesenchymal origin. Immunofluorescence analysis of bleomycin-injured young and aged mouse lungs (60 days post bleomycin) shows that hypoxia, detected by hypoxyprobe, was primarily sensed in PDPN+ AT1 cells and VIM+ mesenchymal cells.

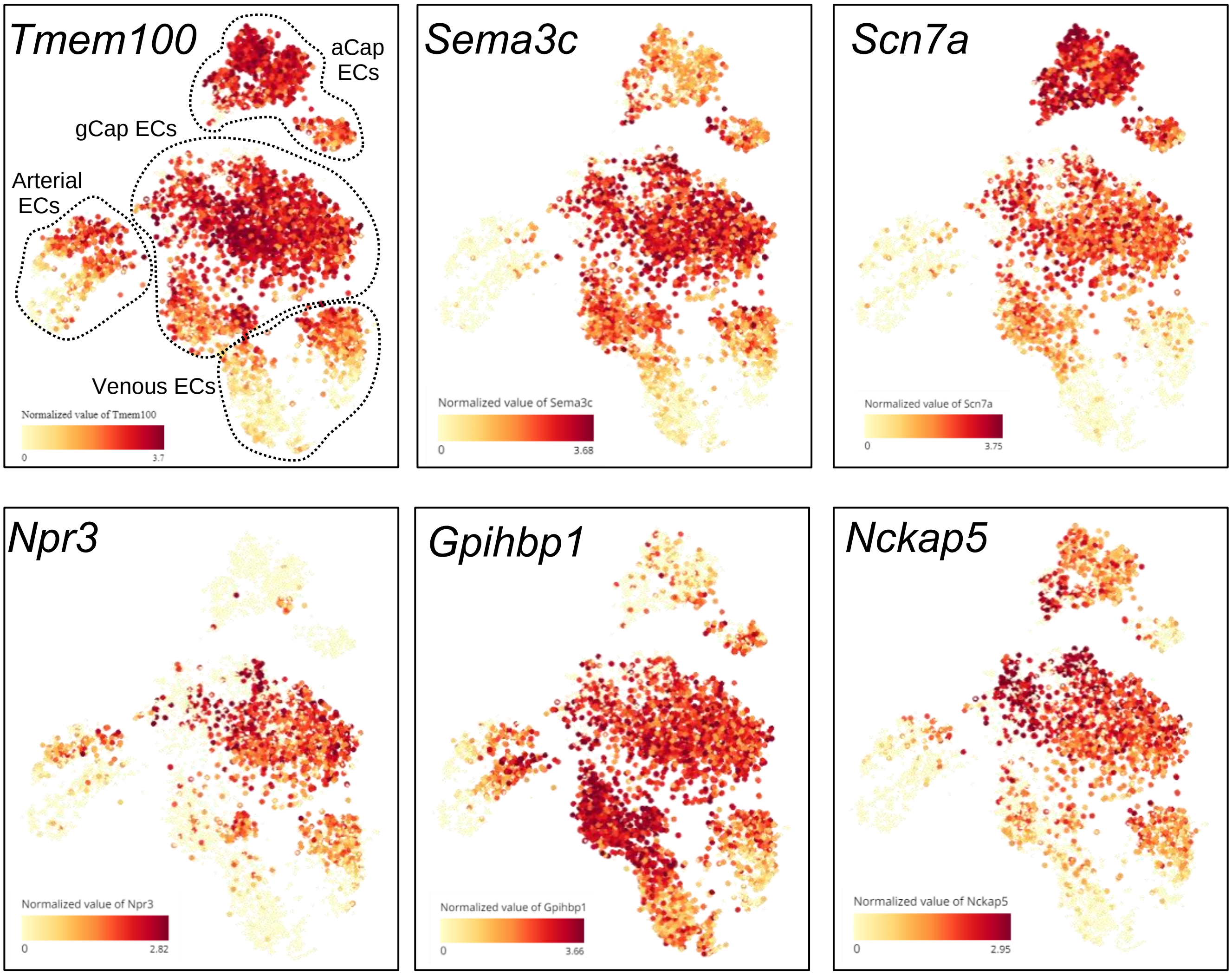

Supplementary Figure 6. GFP+ arterial and venous derived ECs express markers of gCap ECs. t-SNE plots shows the expression of different capillary EC marker genes in GFP+ arterial and venous derived ECs.

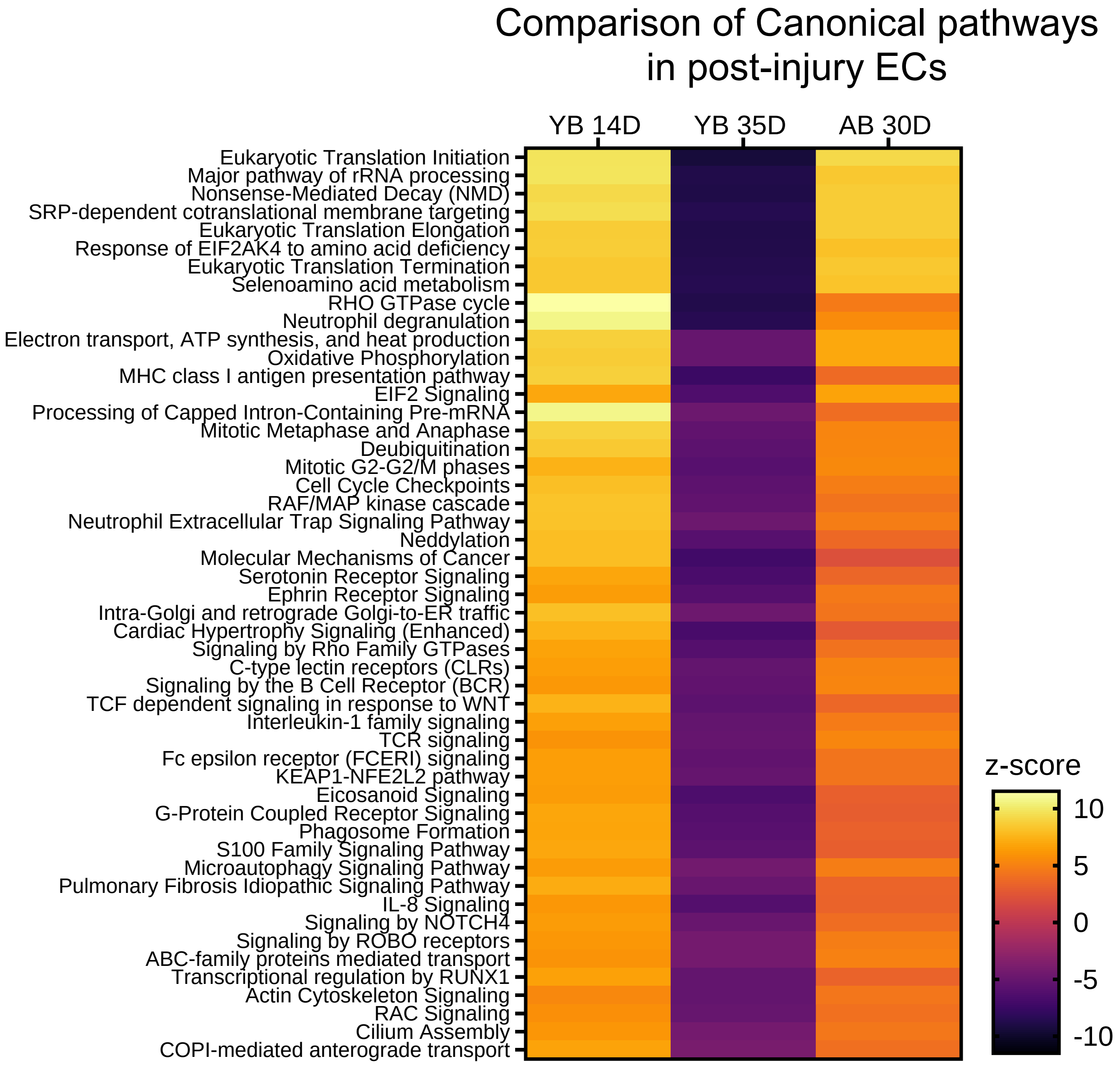

Supplementary Figure 7. Ingenuity pathway analysis shows canonical pathways enriched in lung ECs from young and aged lungs at different timepoints. The same pathway that are deactivated in young lung ECs during the early phase of fibrosis resolution (day 35) post bleomycin challenge remained active in ECs from aged lungs with persistent fibrosis. Activation z-scores for dysregulated genes (Log2 FC ≤ -0.1 or ≥ 0.1, *P* value < 0.05) and comparison analysis were generated in IPA software.

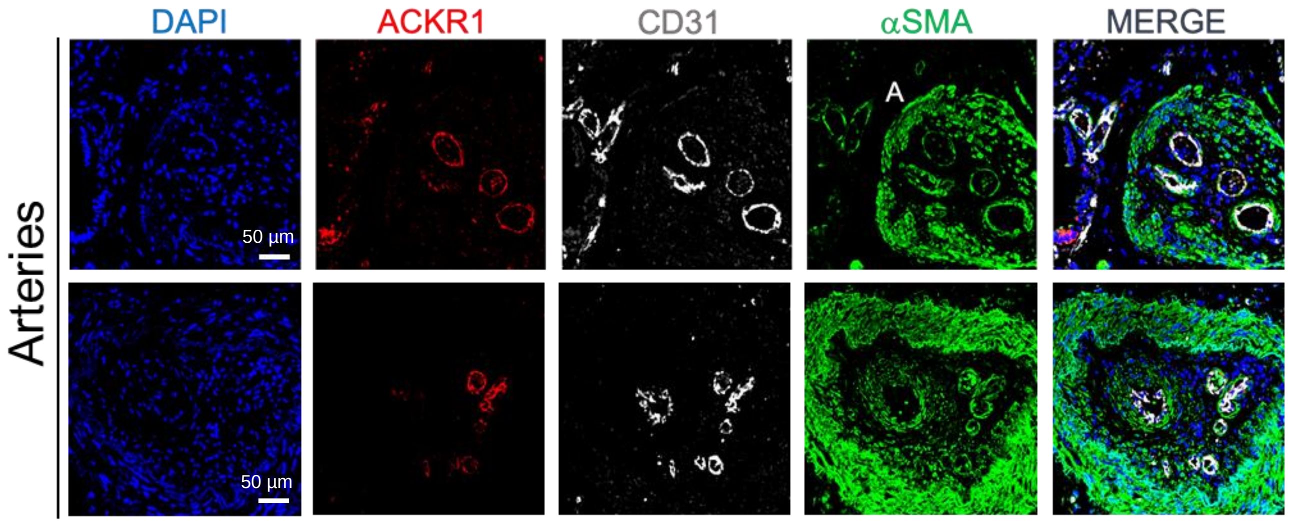

Supplementary Figure 8. Vascular abnormalities involving ACKR1+ venous ECs in IPF lungs. ACKR1+ CD31+ venous ECs were found in the fibrotic intima of numerous arteries (A). Medial thickening and αSMA+ cells within the thickened intima were also observed in these abnormal vessels.

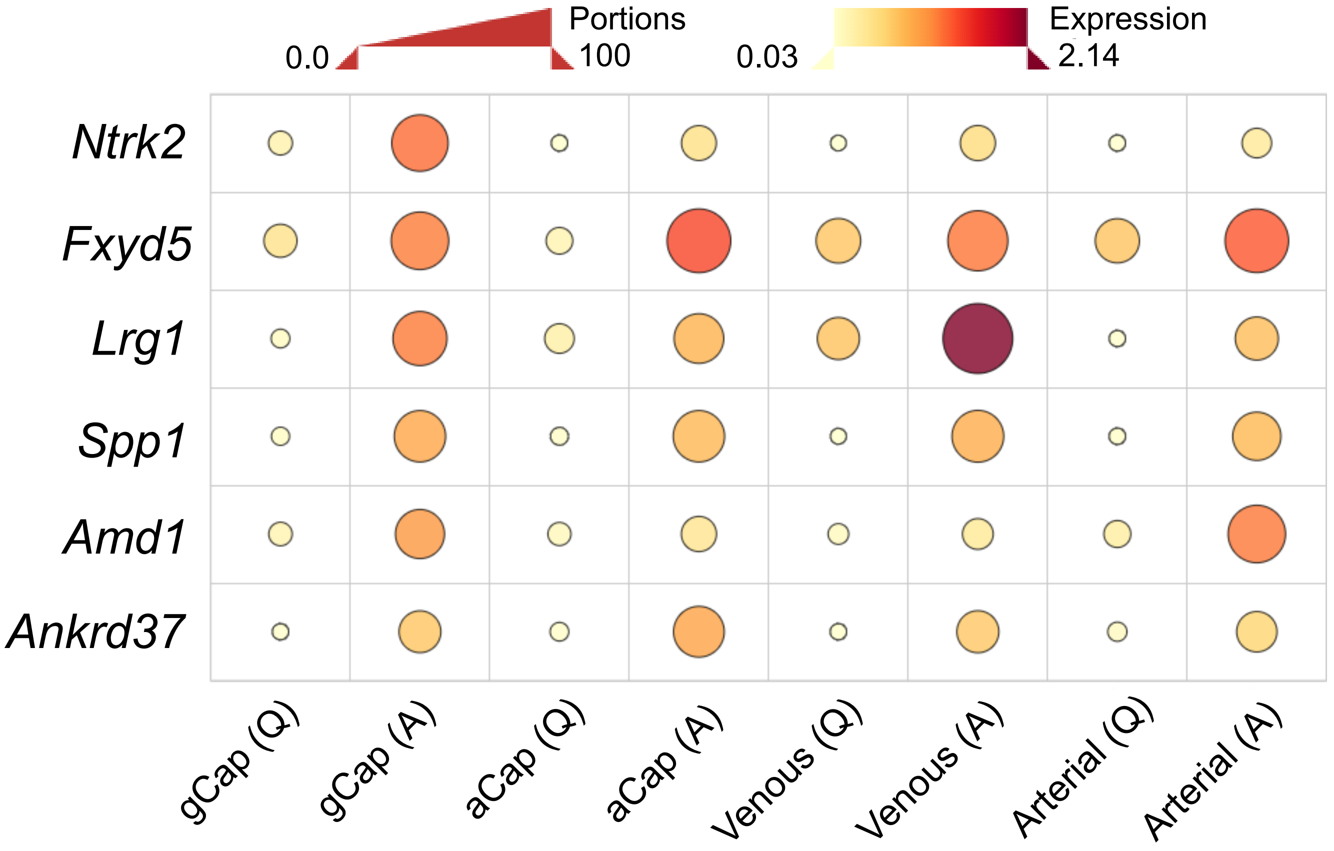

Supplementary Figure 9. *Ntrk2* is mainly expressed in gCap ECs in response to lung injury. Dot plot showing differentially expressed activation marker genes in different EC subpopulations. Dot size indicates the proportion of expressing cells, colored by standardized expression levels. Quiescent (Q), activated (A). aCap (Q, *n* = 1283 cells), aCap (A, *n* = 876 cells), gCap (Q, *n* = 10,256 cells), gCap (A, *n* = 8545 cells), arterial (Q, *n* = 331 cells), arterial (A, *n* = 231 cells), venous (Q, *n* = 959 cells) and venous (A, *n* = 1002 cells).

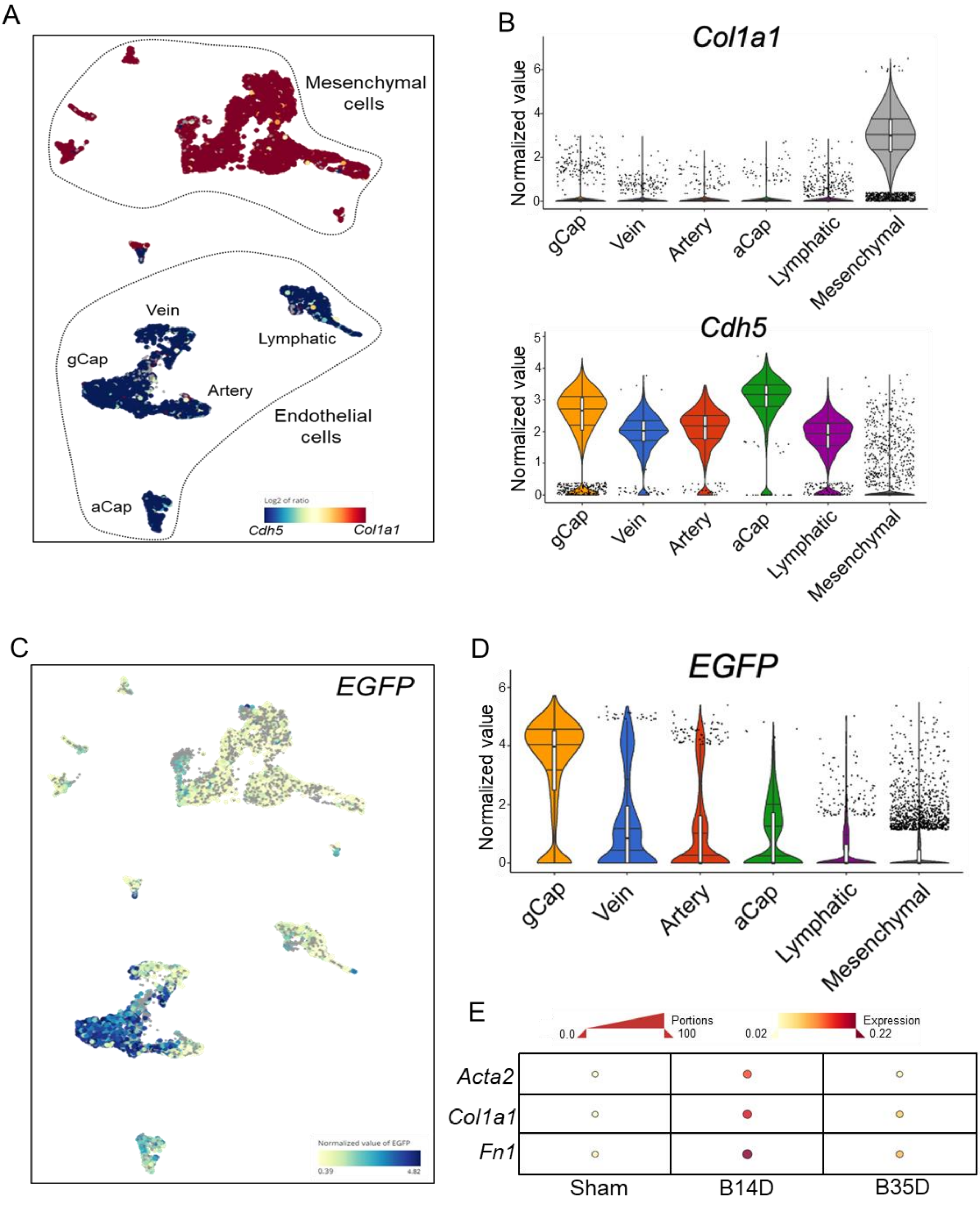
A
Mesenchymal cells
Vein
Lymphatic
gCap
Artery
Endothelial cells
aCap
Log2 of ratio
Cdh5
Col1a1
B
Col1a1
Normalized value
gCap
Vein
Artery
aCap
Lymphatic
Mesenchymal
Cdh5
C
EGFP
Normalized value of EGFP
0.39
4.82
D
EGFP
E
Portions
0.0
100
Expression
0.02
0.22
Acta2
Col1a1
Fn1
Sham
B14D
B35D

Supplementary Figure 10. Capillary ECs do not acquire mesenchymal features during the progression of lung fibrosis. (A, B) UMAP and violins plot show the expression of *Col1a1* and *cdh5* in mesenchymal cells (*n* = 10294 cells) and ECs (*n* = 8057 cells) populations. **(C,D)** UMAP and violin plots shows the expression of *EGFP* in mesenchymal cells and ECs populations. (E) Dot plot showing the expression of *Acta2, Col1a1 and Fn1* in gCap ECs in sham and injured lungs after 14 days (B14D), and 35 days (B35D) post bleomycin-induced lung injury. Dot size indicates the proportion of expressing cells, colored by standardized expression levels. Each box plot displays the median value as the center line, the upper and lower box boundaries at the first and third quartiles (25th and 75th percentiles), and the whiskers depict the minimum and maximum values.

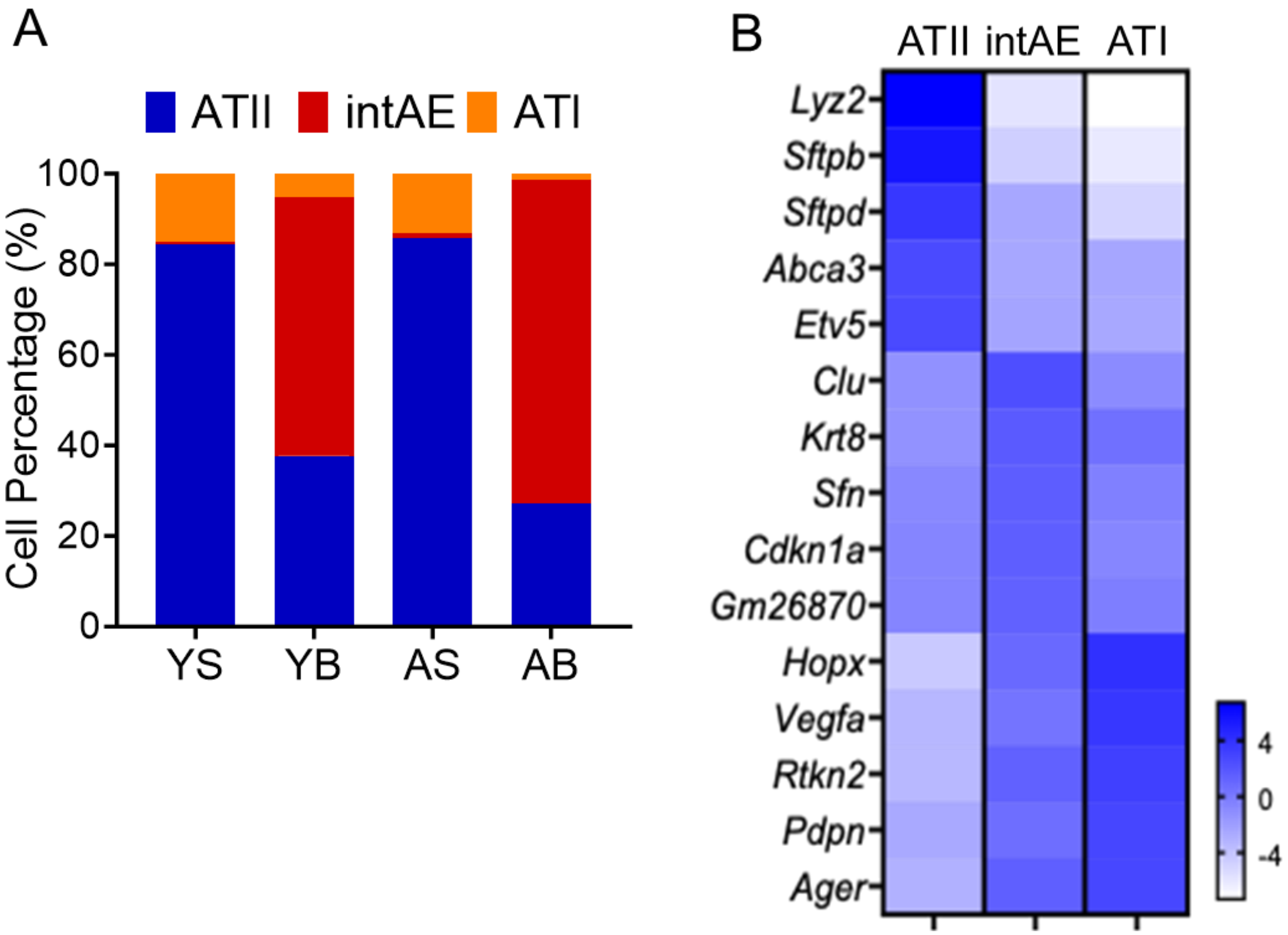

Supplementary Figure 11. Composition and gene marker features of alveolar epithelial cells. (A) Percentage of alveolar epithelial subsets grouped by age and injury. **(B)** Heatmap showing the average expression level of marker genes across different alveolar epithelial cell clusters. Source data are provided as a Source Data file.

Supplementary Table 1. Human primer sequences for qPCR analysis.

Gene	Direction	Sequence (5'-3')
**KIT**	Forward	CGTTCTGCTCCTACTGCTTCG
	Reverse	CCCACGCGGACTATTAAGTCT
**NTRK2**	Forward	TCGTGGCATTTCCGAGATTGG
	Reverse	TCGTCAGTTTGTTTCGGGTAAA
**PLVAP**	Forward	GCTGCTGGTATTACCTGCG
	Reverse	GCCATAGACCATGAAGAGCAC
**TEK**	Forward	TTAGCCAGCTTAGTTCTCTGTGG
	Reverse	AGCATCAGATACAAGAGGTAGGG
**RPLP0**	Forward	AGCCCAGAACACTGGTCTC
	Reverse	ACTCAGGATTTCAATGGTGCC
