# Supplementary material for: Lung injury-induced activated endothelial cell states persist in aging-associated progressive fibrosis
Source: Nat Commun. 2024 Jun 27;15:5449. doi: 10.1038/s41467-024-49545-x (PMC11211333; doi:10.1038/s41467-024-49545-x)
Supplement: Supplementary file 3 — Reporting Summary [file 41467_2024_49545_MOESM3_ESM.pdf]

nature portfolio

Corresponding author(s): Giovanni Ligresti

Last updated by author(s): May 30, 2024

Reporting Summary

Nature Portfolio wishes to improve the reproducibility of the work that we publish. This form provides structure for consistency and transparency in reporting. For further information on Nature Portfolio policies, see our Editorial Policies and the Editorial Policy Checklist.

Statistics

For all statistical analyses, confirm that the following items are present in the figure legend, table legend, main text, or Methods section.

n/a	Confirmed	
☐	☒	The exact sample size (*n*) for each experimental group/condition, given as a discrete number and unit of measurement
☐	☒	A statement on whether measurements were taken from distinct samples or whether the same sample was measured repeatedly
☐	☒	The statistical test(s) used AND whether they are one- or two-sided<br>*Only common tests should be described solely by name; describe more complex techniques in the Methods section.*
☐	☒	A description of all covariates tested
☐	☒	A description of any assumptions or corrections, such as tests of normality and adjustment for multiple comparisons
☐	☒	A full description of the statistical parameters including central tendency (e.g. means) or other basic estimates (e.g. regression coefficient) AND variation (e.g. standard deviation) or associated estimates of uncertainty (e.g. confidence intervals)
☐	☒	For null hypothesis testing, the test statistic (e.g. *F*, *t*, *r*) with confidence intervals, effect sizes, degrees of freedom and *P* value noted<br>*Give P values as exact values whenever suitable.*
☒	☐	For Bayesian analysis, information on the choice of priors and Markov chain Monte Carlo settings
☒	☐	For hierarchical and complex designs, identification of the appropriate level for tests and full reporting of outcomes
☒	☐	Estimates of effect sizes (e.g. Cohen's *d*, Pearson's *r*), indicating how they were calculated

Our web collection on statistics for biologists contains articles on many of the points above.

Software and code

Policy information about availability of computer code

Data collection	Data were collected on the following instruments: Illumina NextSeq 500, Illumina NextSeq 2000, Zeiss LSM 700, Zeiss Axio Scan.Z1, Olympus BH-2 microscope, Olympus CKX53 Microscope,BD LSR II (BD Biosciences) Step-One-Plus Real-Time PCR system (Applied Biosystems), (GSE136831)
Data analysis	The following software and tools were used for data analysis: BioTuring Browser 3, Ingenuity Pathway analysis (IPA, Ingenuity® Systems, www.ingenuity.com), Graphpad Prism 8.4.3 (la Jolla, CA, USA), FlowJo software version 10.8.0, ImageJ, Venny 2.0 (https://bioinfogp.cnb.csic.es/tools/venny/index2.0.2.html), iVision-Mac 4.5.4, ZEN 2.3 SP1 FP3 Black, Zeiss ZEN 3.1 Blue, INFINITY ANALYZE 6.5.4, R arm64 release for macOS 11 with RStudio as the integrated development environment (IDE).

For manuscripts utilizing custom algorithms or software that are central to the research but not yet described in published literature, software must be made available to editors and reviewers. We strongly encourage code deposition in a community repository (e.g. GitHub). See the Nature Portfolio guidelines for submitting code & software for further information.

Data

Policy information about availability of data

All manuscripts must include a data availability statement. This statement should provide the following information, where applicable:

- Accession codes, unique identifiers, or web links for publicly available datasets
- A description of any restrictions on data availability
- For clinical datasets or third party data, please ensure that the statement adheres to our policy

The scRNA-seq data generated in this study have been deposited in the Gene Expression Omnibus under the GEO accession numbers GSE264151 and GSE264162. The publicly available scRNA-seq dataset from human IPF and healthy lungs used in this study are available in the GEO database under accession code GSE136831. All the remaining data are available within the Article, Supplementary Information or Source Data file. Source data are provided with this paper.

Research involving human participants, their data, or biological material

Policy information about studies with human participants or human data. See also policy information about sex, gender (identity/presentation), and sexual orientation and race, ethnicity and racism.

Reporting on sex and gender	Sex and/or gender was not considered in the study design. Both sexes were included in the study and distributed randomly in the experiments, and we did not observe any sex-dependent differences in our findings. Due to the limited number of patient samples used in the analysis, statistical adjustment for sex or gender analysis was not performed. In this study, we used lung tissues from five normal donors (two females and three males) and nine patients with IPF (four females and five males).
Reporting on race, ethnicity, or other socially relevant groupings	Race and ethnicity were not link to the study.
Population characteristics	N/A
Recruitment	N/A
Ethics oversight	Lung tissues from patients with IPF were obtained from explanted lungs obtained at the time of transplantation. All patients provided written informed consent and the study was approved by the University of Michigan Institutional Review Board, Ann Arbor, MI (HUM00105694). Diagnoses of patients with IPF were established by clinical pathological criteria and confirmed by multidisciplinary consensus conference. Additional IPF lung tissue was obtained from a deceased individual; permission for the autopsy on this case was granted by the next of kin (VA Medical Center, Seattle, WA). Normal control lungs were obtained from deceased donors whose lungs were deemed unsuitable for transplant and were provided by Gift of Life, Michigan, with consent from family for tissue to be used for research purposes. No compensation was provided to subjects or family for either IPF patient samples or normal control lungs.

Note that full information on the approval of the study protocol must also be provided in the manuscript.

Field-specific reporting

Please select the one below that is the best fit for your research. If you are not sure, read the appropriate sections before making your selection.

☒ Life sciences ☐ Behavioural & social sciences ☐ Ecological, evolutionary & environmental sciences

For a reference copy of the document with all sections, see nature.com/documents/nr-reporting-summary-flat.pdf

Life sciences study design

All studies must disclose on these points even when the disclosure is negative.

Sample size	Sample sizes were determined based on prior studies performed in the field and the number of replicates for statistical analyses are specified in the figure legends.
Data exclusions	No data were excluded from the experiments.
Replication	The bleomycin experiments performed with WT and different transgenic mice were all confirmed with successful replication, with different cohorts and with consistent data. Figure legends indicate the number of mice (for in vivo experiments) and the number of biologically independent replicates (for in vitro experiments)
Randomization	Randomization was not relevant for this study. Animals were assigned into experimental groups based on their desired genotypes ,if it is needed, for comparison. Efforts were made to confirm the phenotypes across mice from multiple different litters and cages.
Blinding	Investigators were not blinded to group allocation as they had to be aware of the groups during separation and treatment steps. Blinding was

Blinding

not used when analyzing results from scRNA-seq due to the unbiased nature of the bioinformatics approaches used to quantify differential expression between age and treatment.

Reporting for specific materials, systems and methods

We require information from authors about some types of materials, experimental systems and methods used in many studies. Here, indicate whether each material, system or method listed is relevant to your study. If you are not sure if a list item applies to your research, read the appropriate section before selecting a response.

Materials & experimental systems

n/a	Involved in the study
☐	☒ Antibodies
☐	☒ Eukaryotic cell lines
☒	☐ Palaeontology and archaeology
☐	☒ Animals and other organisms
☒	☐ Clinical data
☒	☐ Dual use research of concern
☒	☐ Plants

Methods

n/a	Involved in the study
☒	☐ ChIP-seq
☐	☒ Flow cytometry
☒	☐ MRI-based neuroimaging

Antibodies

Antibodies used

anti-CD31 rat antibody (550274, clone MEC 13.3, BD Biosciences, San Jose, CA, USA, 1:200 dilution), anti- CD31mouse antibody (131M-94, clone JC70, Cell Marque, Millipore Sigma, USA, 1:200 dilution), anti-TrkB rabbit antibody (4607S, clone 80G2, Cell Signaling Technology, Danvers, MA, USA 1:200 dilution), anti-C1qR1/CD93 goat antibody (AF2379, R&D systems, Minneapolis, MN, USA 1:200 dilution).
Immunofluorescence staining
anti-CD31 rabbit antibody (77699, clone D8V9E, Cell Signaling Technology, Danvers, MA, USA 1:200 dilution), anti-TrkB goat antibody (AF1494, Novus, Centennial, CO, USA, 1:200 dilution), anti-CAR4 goat antibody (AF2414, R&D systems, Minneapolis, MN, USA 1:200 dilution), anti-αSMA mouse antibody (F3777, clone 1A4, Sigma-Aldrich, St. Louis, MA, USA, 1:200 dilution), anti-mouse ACKR1 rat antibody (kindly provided by Dr. von Andrian, 1:200 dilution), anti-CD31 mouse antibody (3528, clone 89C2, Cell Signaling Technology, Danvers, MA, USA 1:200 dilution), anti-ACKR1 goat antibody (NB100-2421, Novus, Centennial, CO, USA, 1:200 dilution), anti-Col1α1 antibody (72026, clone E8F4L, Cell Signaling Technology, Danvers, MA, USA 1:200 dilution), Anti-PDPN Syrian hamster antibody (13-5381-82, Clone eBio8.1.1 (8.1.1), Thermo Fisher Scientific, Waltham, MA, USA, 1:300 dilution), and anti-Vimentin rabbit antibody (5741S, clone D21H3, Cell Signaling Technology, Danvers, MA, USA 1:300 dilution), Hypoxyprobe™-1 RED PE (HP-1 RED PE Mab-1, Hypoxyprobe, Inc; Burlington, MA, USA, 1:100 dilution), donkey anti-Goat-555 (A32816, Thermo Fisher Scientific, Waltham, MA, USA,1:1000 dilution), donkey anti-Mouse-488 (A21202, Thermo Fisher Scientific, 1:1000 dilution), donkey anti-Mouse-647 (A31571, Thermo Fisher Scientific, 1:1000 dilution), donkey anti-Rabbit-488 (A21206, Thermo Fisher Scientific, 1:1000 dilution), donkey anti-Rabbit-647 (A31573, Thermo Fisher Scientific, 1:1000 dilution), donkey anti-Rat-488 (A21208, Thermo Fisher Scientific, 1:1000 dilution), goat anti-Syrian hamster-488 (107-546-142, Jackson ImmunoResearch, West Grove, PA, USA, 1:1000 dilution)
FACS analysis
antiCD45:Pacific blue (368539, clone 2D1, Biolegend, San Diego, CA, USA, 1:200 dilution), anti-EpCAM:BV650 (324225, colon 9C4, Biolegend, San Diego, CA, USA, 1:200 dilution), anti-CD31:APC/Cyanine7 (303119, clone WM59, Biolegend, San Diego, CA, USA, 1:200 dilution), anti-Tek:PE (334205, clone 33.1, Biolegend, San Diego, CA, USA, 1:100 dilution), anti-ACKR1 (NB100-2421, Novus, Centennial, CO, USA, 1:50 dilution), anti-P-Selectin (NB100-65392, Novus, Centennial, CO, USA, 1:50 dilution), anti-CTHRC1 (PA5-49638, Invitrogen, Waltham, MA, USA, 1:50 dilution), anti-rabbit IgG:FITC (406403, clone Poly4064, Biolegend, San Diego, CA, USA, 1:200 dilution), anti-goat IgG:PE (405307, clone Poly4053 , Biolegend, San Diego, CA, USA, 1:200 dilution)

Validation

All antibodies were commercially available and were validated by the respective manufactures. Additional information can be found at the following links:
Cell signaling Technology: https://www.cellsignal.com/about-us/cst-antibody-validation-principles
At Cell Signaling Technology (CST), we understand that there is no single assay that can determine the validity of an antibody. Confirming that an immunoreagent is sufficiently specific and sensitive depends on the application and protocol being used, the type and quality of sample being analyzed, and the inherent biophysical properties of the antibody itself.
To ensure our antibodies will work in your experiment, we adhere to the Hallmarks of Antibody Validation™, six complementary strategies that can be used to determine the functionality, specificity, and sensitivity of an antibody in any given assay. CST adapted the work by Uhlen, et. al., ("A Proposal for Validation of Antibodies." Nature Methods (2016)) to build the Hallmarks of Antibody Validation, based on our decades of experience as an antibody manufacturer and our dedication to reproducible science.

ThermoFisher: https://www.thermofisher.com/us/en/home/life-science/antibodies/invitrogen-antibody-validation.html
At Thermo Fisher Scientific, we help provide confidence in selecting the right antibody for the intended research needs to enable researchers to successfully reproduce and confirm experimental results. This means that an antibody must be specific and selective within the context it is being used. To achieve this, we use a comprehensive approach to antibody verification that is tailored to the antibody target and the relevant application. Our advanced verification occurs in addition to standard antibody testing that happens during manufacturing.

BD Bioscience: https://www.bdbiosciences.com/en-us/products/reagents/flow-cytometry-reagents/research-reagents/quality-and-reproducibility

BD Biosciences identifies key targets of interest in scientific research and develops its own specific antibodies or collaborates with top research scientists around the world to license their antibodies. We then transform these antibodies into flow cytometry reagents by conjugating them to a broad portfolio of high-performing dyes, including our vastly popular portfolio of BD Horizon Brilliant™ Dyes. A world-class team of research scientists helps ensure that these reagents work reliably and consistently for flow cytometry applications. The specificity is confirmed using multiple methodologies that may include a combination of flow cytometry, immunofluorescence, immunohistochemistry or western blot to test staining on a combination of primary cells, cell lines or transfectant models.

Millipore/Sigma: https://www.sigmaaldrich.com/US/en/technical-documents/technical-article/protein-biology/elisa/antibody-standard-validation
The success of any immunodetection experiment depends on the quality of the antibodies which are employed. However, antibody reagents vary significantly and when selecting an antibody for a downstream application it is a good idea to spend some time ensuring that not only has it been tested in the chosen experimental setup but that it also demonstrates the required specificity, sensitivity and reproducibility. This valuable data is generated during the antibody manufacturing and validation process and can be found on the product datasheet with which the antibody is supplied.

Novus: https://www.novusbio.com/reproducibility.html
Novus recognizes the need for highly validated, high quality antibodies in the life sciences community. The research community faces ongoing concerns about data reproducibility and especially the validity of antibody-based assays. A recent article in Nature discusses the variable standards and performance of antibodies and antibody suppliers in the market. Novus is committed to addressing this problem and to helping our customers attain the best possible results with our products.
To that end, we actively seek high quality, highly validated products and provide support to ensure that our customers have the tools to properly validate their own assays. We are also collaborating with several global initiatives that help life science researchers choose antibodies with proven results. Of the five pillars of validation established by these initiatives, genetic knockout validation provides the most reliable control for assessing antibody specificity. For technical support in validating your antibody based applications, visit technical support.

Biolegend: https://www.biolegend.com/en-us/bio-bits/highly-specific-validated-antibodies
Antibody validation is a critical step in the journey towards obtaining consistent reproducibility in science. To ensure they are both specific and sensitive, we validate our antibodies through a variety of methods including:
Testing on multiple cell and tissue types with a variety of known expression levels.
Validation in multiple applications as a cross-check for specificity and to provide additional clarity for researchers.
Comparison to existing antibody clones.
Using cell treatments to modulate target expression, such as phosphatase treatment to ensure phospho-antibody specificity.

R&D systems: https://www.rndsystems.com/quality/antibodies-built-for-reproducibility
With the recent reports stating antibodies as one of the reasons for scientific irreproducibility, you can rest assured with our antibodies. R&D Systems® takes rigorous steps towards antibody validation and reproducibility. We have been since the beginning. For 30 years, we have used our industry-leading production standards and quality control specifications to develop antibodies that can be relied on for specificity and reproducibility. By developing and testing our products in-house, we can ensure a validated and specific antibody. We are confident in our antibodies and provide 100% guarantee for our products. With R&D Systems® antibodies your results will stand the test of time.

Eukaryotic cell lines

Policy information about cell lines and Sex and Gender in Research

Cell line source(s)	Human lung microvascular endothelial cells (HLMECs) were purchased from Cell Applications (San Diego, CA, USA) or ANGIO-PROTEOMIE (Worcester, MA, USA).
Authentication	cell lines used were not authenticated
Mycoplasma contamination	cell lines tested negative
Commonly misidentified lines (See ICLAC register)	No commonly misidentified lines were used

Animals and other research organisms

Policy information about studies involving animals; ARRIVE guidelines recommended for reporting animal research, and Sex and Gender in Research

Laboratory animals	Male young (2 months old) and aged (18 months old) C57BL/6J mice were purchased from Jackson Laboratory (Bar Harbor, ME). Male and female Col1a1-GFP transgenic mice (2 months old, FVB strain) were generated as previously described (UC San Diego, La Jolla, CA)90 and kindly provided by Dr. Derek Radisky. Male and female (2 months old) Aplnr-Cre-ER(T):Rosa26-mdtTomato/mGFP reporter mice on a C57/Bl6 background were generated by breeding Aplnr-CreER (Tg(Aplnr-cre/ERT2)91 (Kindly provided by Dr. Kristy Red-Horse, Stanford University) and B6.129(Cg) – Gt(ROSA)26Sortm4(ACTB-tdTomato,-EGFP)Luo/J (Strain #007676, Jackson Laboratory, Bar Harbor, ME).
Wild animals	no wild animals were used
Reporting on sex	All experiments were conducted using a mixture of male and female mice, except for the scRNA-seq experiment focusing on aging, which was specifically performed using male mice. This decision was based on the observation that human fibrosis is an age-

	associated disease, with approximately 70% of idiopathic cases occurring in males. Therefore, the rationale for utilizing male animals in this particular study was to better model the age-associated nature of the disease and its prevalence in males.
Field-collected samples	no field-collected samples were used
Ethics oversight	All animal experiments were done in accordance with protocols approved by the Institutional Animal Care and Use Committee at Boston University.

Note that full information on the approval of the study protocol must also be provided in the manuscript.

Plants

Seed stocks	N/A
Novel plant genotypes	N/A
Authentication	N/A

Flow Cytometry

Plots

Confirm that:

☒ The axis labels state the marker and fluorochrome used (e.g. CD4-FITC).

☒ The axis scales are clearly visible. Include numbers along axes only for bottom left plot of group (a 'group' is an analysis of identical markers).

☒ All plots are contour plots with outliers or pseudocolor plots.

☒ A numerical value for number of cells or percentage (with statistics) is provided.

Methodology

Sample preparation	As described in Methods, human normal and IPF lungs were minced with a razor blade in a 100 mm petri dish in a cold DMEM medium containing 0.2 mg/ml Liberase DL and 100 U/ml DNase I (Roche, Indianapolis, IN, USA). The mixture was transferred into 15 ml tubes and incubated at 37 °C for 35 min in a water bath under continuous rotation to allow enzymatic digestion. Digestion was inactivated with a DMEM medium containing 10% fetal bovine serum, the cell suspension was passed through a 40 µm cell strainer (Fisher, Waltham, MA, USA) to remove debris. Cells were then centrifuged (500×g, 10 min, 4 °C), and resuspended in 3 ml red blood cell lysis buffer (Biolegend, San Diego, CA, USA) for 90 s to remove the remaining red blood cells and diluted in 9 mL PBS after incubation. Cells were then centrifuged (500×g, 10 min, 4 °C) and resuspended in 0.2 ml of FACS buffer (0.05% BSA, 0.5 mM EDTA pH 7.4 in PBS).
Instrument	BD LSR II (BD Biosciences, San Jose, CA, USA).
Software	Data were analyzed in Flowjo 10.8.0
Cell population abundance	No tests were performed to asses the purity of each population after sorting
Gating strategy	FACS analysis was performed with the following strategy: debris exclusion (FSC-A by SSC-A), doublet exclusion (SSC-W by SSC-H and FSC-W by FSC-H), and dead cell exclusion (DAPI by FSC-A).

☐ Tick this box to confirm that a figure exemplifying the gating strategy is provided in the Supplementary Information.
